# A guide to selecting high-performing antibodies for human Midkine for use in Western blot and immunoprecipitation

**DOI:** 10.12688/f1000research.130587.3

**Published:** 2024-07-23

**Authors:** Riham Ayoubi, Kathleen Southern, Carl Laflamme, NeuroSGC/YCharOS Collaborative Group

**Affiliations:** 1 Department of Neurology and Neurosurgery, Structural Genomics Consortium, The Montreal Neurological Institute, McGill University, Montreal, Quebec, Canada

**Keywords:** Uniprot #P21741, MDK, Midkine, antibody validation, antibody characterization, Western blot, immunoprecipitation

## Abstract

Midkine is a secreted protein that acts as a growth factor or cytokine involved in cell survival and inflammatory processes. It accumulates in amyloid plaques, which are hallmarks of Alzheimer’s Disease (AD). The reproducibility of Midkine research would be enhanced if the community had access to well-characterized anti-Midkine antibodies. In this study, we characterized 8 commercial Midkine antibodies for Western blot and immunoprecipitation, using a standardized experimental protocol based on comparing read-outs in a knockout cell line and isogenic parental control. These studies are part of a larger, collaborative initiative seeking to address the antibody reproducibility issue by characterizing commercially available antibodies for human proteins and publishing the results openly as a resource for the scientific community. While use of antibodies and protocols vary between laboratories, we encourage readers to use this report as a guide to select the most appropriate antibodies for their specific needs.

## Introduction

The neurotrophic and developmental factor Midkine is a secreted heparin-binding cytokine. ^1^ It mediates its diverse physiological functions by binding to cell-surface proteoglycan receptors via their chondroitin sulfate groups, thereby regulating cell proliferation, migration and differentiation. ^2^^–^^4^

Midkine is involved in numerous processes that promote cell growth, and may contribute to the pathogenesis of inflammatory diseases. ^1^^,^^4^ Proteomic analyses have found that Midkine is highly enriched in amyloid-beta plaques, indicating its potential as a biomarker and/or therapeutic target for Alzheimer’s Disease (AD). ^5^^–^^7^ Mechanistic studies would be greatly facilitated by the availability of high-quality antibodies for Midkine.

This research is part of a broader collaborative initiative in which academics, funders and commercial antibody manufacturers are working together to address antibody reproducibility issues by characterizing commercial antibodies for human proteins using standardized protocols, and openly sharing the data. ^8^^–^^10^ Here, we compared the performance of a range of commercially available antibodies for Midkine use in Western Blot and immunoprecipitation. This article serves as a valuable guide to help researchers select high-quality antibodies for their specific needs, facilitating the biochemical and cellular assessment of Midkine properties and function.

## Results and discussion

Our standard protocol involves comparing readouts from wild-type and knockout cells. ^11^^–^^13^ The first step is to identify a human cell line(s) that expresses sufficient levels of a given protein to generate a measurable signal. To this end, we examined the DepMap transcriptomics database to identify all cell lines that express the target at levels greater than 2.5 log2 (transcripts per million “TPM”+1), which we have found to be a suitable cut-off (Cancer Dependency Map Portal, RRID: SCR_017655). Commercially available human HAP1 cells expressed the Midkine transcript at RNA levels above the average range of cancer cells analyzed. ^14^ Parental and *MDK* knockout human HAP1 cells were obtained from Horizon Discovery ( Table 1).

**Table 1.** Summary of the cell lines used.

Institution	Catalog number	RRID (Cellosaurus)	Cell line	Genotype
Horizon Discovery	C631	CVCL_Y019	HAP1	WT
Horizon Discovery	HZGHC007906c008	CVCL_C6Y2	HAP1	*MDK* KO

**Figure 1.**
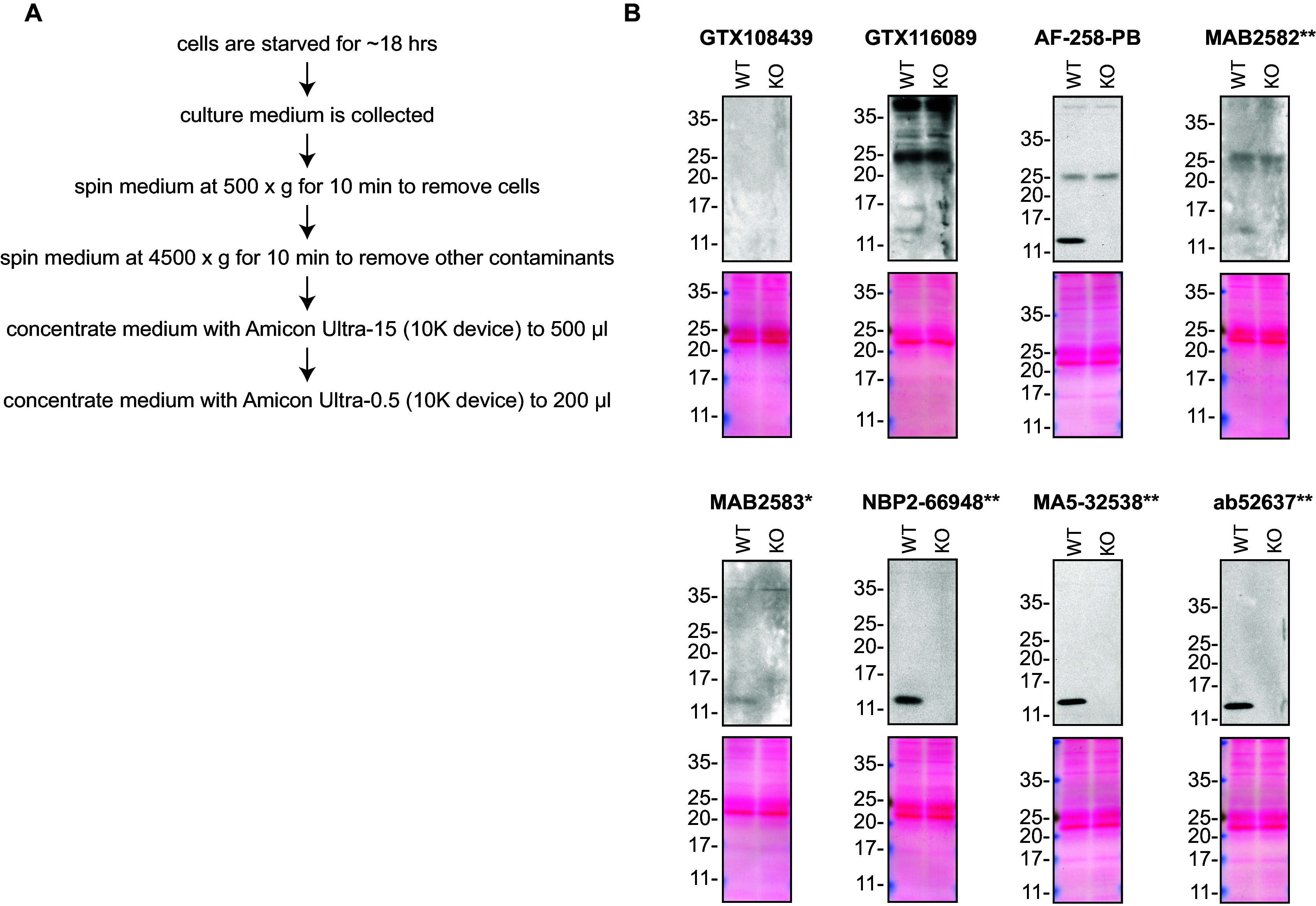
Midkine antibody screening by Western blot on culture media. A) Schematic of the protocol used to concentrate the culture media. B) Lysates of HAP1 (WT and *MDK* KO) were prepared, and ~30 μg of protein was processed for Western blot with the indicated Midkine antibodies. The Ponceau stained transfers of each blot are presented to show equal loading of WT and KO lysates and protein transfer efficiency from the acrylamide gels to the nitrocellulose membrane. Antibody dilutions were chosen according to the recommendations of the antibody supplier. Exceptions were given for antibodies GTX116089 and ab52637**, which were titrated to 1/1000 and 1/500, respectively, as the signal was too weak when following the supplier’s recommendations. When suppliers did not recommend dilutions, we tested the antibodies at 1/200. Antibody dilution used: GTX108439 at 1/1000; GTX116089 at 1/100; AF-258-PB at 1/100; MAB2582** at 1/200; MAB2583* at 1/200; NBP2-66948** at 1/500; MA5-32538** at 1/500; ab52637** at 1/500. Predicted band size: 15 kDa. *Monoclonal antibody, **Recombinant antibody.

**Figure 2.**
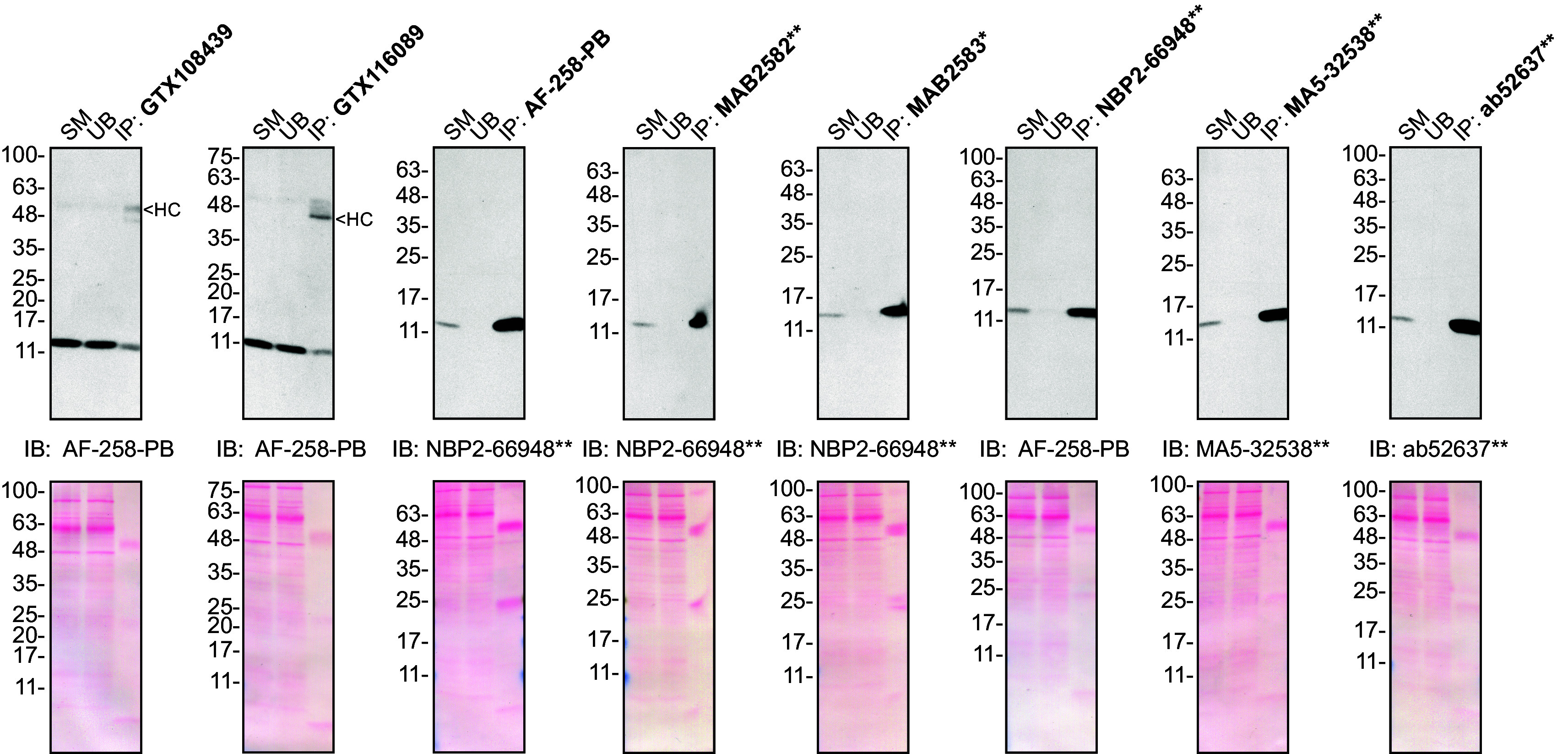
Midkine antibody screening by immunoprecipitation on culture media. Concentrated culture media were prepared as in 1A). Immunoprecipitation was performed using 1.0 μg of the indicated Midkine antibodies pre-coupled to either protein A or protein G magnetic beads. Samples were washed and processed for Western blot with the indicated Midkine antibody. For Western blot, AF-258-PB was used at 1/500, NBP2-66948** at 1/500, MA5-32538** at 1/200 and ab52637** at 1/200. The Ponceau stained transfers of each blot are shown. SM = 10% starting material; UB = 10% unbound fraction; IP = immunoprecipitate; HC = antibody heavy chain. *Monoclonal antibody, **Recombinant antibody.

Midkine is predicted to be a secreted protein. Accordingly, we collected concentrated culture media from both wild-type and *MDK* KO cells and used the conditioned media to probe the performance of the antibodies ( Table 2) side-by-side by Western blot and immunoprecipitation. The profiles of the tested antibodies are shown in Figures 1 and 2.

**Table 2.** Summary of the Midkine antibodies tested.

Company	Catalog number	Lot number	RRID (Antibody Registry)	Clonality	Clone ID	Host	Concentration (μg/μl)	Initial vendors recommended applications
GeneTex	GTX108439	39855	AB_1950903	polyclonal	-	rabbit	0.33	Wb
GeneTex	GTX116089 ^a^	40597	AB_11165850	polyclonal	-	rabbit	1.00	Wb
Bio-Techne	AF-258-PB	WE0519091	AB_2143400	polyclonal	-	goat	0.20	Wb
Bio-Techne	MAB2582 **	CMHR0219071	AB_2893288	recombinant-mono	1011522	mouse	0.50	IF
Bio-Techne	MAB2583 *	CMLT0120031	AB_2893289	monoclonal	1011622	mouse	0.50	-
Bio-Techne	NBP2-66948 ** ^a^	HN0907	AB_2893290	recombinant-mono	JF096-5	rabbit	1.00	Wb, IP
Thermo	MA5-32538 **	WD3265256	AB_2809815	recombinant-mono	JF096-5	rabbit	1.00	Wb
Abcam	ab52637 **	GR3315059-2	AB_880698	recombinant-mono	EP1143Y	rabbit	0.10	Wb, IP, IF

Wb = Western blot; IF = immunofluorescence; IP = immunoprecipitation.

* Monoclonal antibody.

** Recombinant antibody.

^a^ Antibodies that have been discontinued subsequent to the release of this study.

In conclusion, we screened eight Midkine commercial antibodies by Western blot and immunoprecipitation. Several high-quality antibodies that successfully detect Midkine under our standardized experimental conditions can be identified. In our effort to address the antibody reliability and reproducibility challenges in scientific research, the authors recommend the antibodies that demonstrated to be underperforming under our standard procedure be removed from the commercial antibody market. However, the authors do not engage in result analysis or offer explicit antibody recommendations. A limitation of this study is the use of universal protocols - any conclusions remain relevant within the confines of the experimental setup and cell line used in this study. Our primary aim is to deliver top-tier data to the scientific community, grounded in Open Science principles. This empowers experts to interpret the characterization data independently, enabling them to make informed choices regarding the most suitable antibodies for their specific experimental needs.

Antibodies GTX 116089 and NBP2-66948 were removed from the commercial antibody market and are no longer available. Antibody MA5-32538 has the same clone ID as NBP2-66948, and is still available on the market as an alternative.

## Methods

### Antibodies

All Midkine antibodies are listed in Table 2. Peroxidase-conjugated goat anti-rabbit, anti-mouse and donkey anti-goat antibodies are from Thermo Fisher Scientific (cat. number 65-6120, 62-6520 and A15999, respectively).

### Cell culture

Cells were cultured in DMEM high-glucose (GE Healthcare cat. number SH30081.01) containing 10% fetal bovine serum (Wisent, cat. number 080450), 2 mM L-glutamate (Wisent cat. number 609065, 100 IU penicillin and 100 μg/ml streptomycin (Wisent cat. number 450201). Cells were starved in DMEM high glucose containing L-glutamate and penicillin/streptomycin.

### Antibody screening by Western blot on culture medium

HAP1 cells (WT and *MDK* KO) were washed 3× with PBS and starved for ~18 hrs. Culture media were collected and centrifuged for 10 min at 500 × g to eliminate cells and larger contaminants, then for 10 min at 4500 × g to eliminate smaller contaminants.

Culture media were initially concentrated using Amicon Ultra-15 Centrifugal Filter Units (MilliporeSigma cat. number UFC9010) by centrifuging at 4000 × g for 15 min. The resulting 500 μl of the concentrated media were centrifuged again at 4000 × g for 15 min using Amicon Ultra- 0.5 Centrifugal Filter Units (MilliporeSigma cat. number UFC5010) to 200 μl.

Western blots were performed as described in our standard operating procedure. ^13^^,^^15^ A Bradford reagent assay was performed to determine the protein concentration of the culture medium. The concentration was then adjusted to ensure equal amounts of protein were loaded in each gel. Western blots were performed with large 10-20% gradient polyacrylamide gels, loaded with 30 μg of protein in each lane and then transferred on nitrocellulose membranes. Proteins on the blots were visualized with Ponceau staining which is scanned to show together with individual Western blot. Blots were blocked with 5% milk for 1 hr, and antibodies were incubated overnight at 4°C with 5% bovine serum albumin in TBS with 0.1% Tween 20 (TBST). Following three washes with TBST, the peroxidase conjugated secondary antibody was incubated at a dilution of ~0.2 μg/ml in TBST with 5% milk for 1 hr at room temperature followed by three more washes with TBST. Membranes were incubated with ECL from Pierce (cat. number 32106) prior to detection with HyBlot CL autoradiography films from Denville (cat. number 1159T41).

### Antibody screening by immunoprecipitation on culture medium

Immunoprecipitation was performed as described in our standard operating procedure. ^13^^,^^16^ Antibody-bead conjugates were prepared by adding 1.0 μg of antibody to 500 μl of Pierce IP Lysis from Thermo Fisher Scientific (cat. number 87788) in a microcentrifuge tube, together with 30 μl of Dynabeads protein A - (for rabbit antibodies) or protein G - (for mouse and goat antibodies) from Thermo Fisher Scientific (cat. number 10002D and 10004D, respectively). Tubes were rocked overnight at 4°C followed by two washes with the Pierce IP Buffer to remove unbound antibodies. Pierce IP Lysis Buffer (25 mM Tris-HCl pH 7.4, 150 mM NaCl, 1 mM EDTA, 1% NP-40 and 5% glycerol) was supplemented with 1× Halt Protease and Phosphatase Inhibitor Cocktail from Thermo Fisher Scientific (cat. number 78446).

Starved HAP1 WT culture media were concentrated as described above. The concentrated culture media were diluted in Pierce IP Lysis Buffer, and 1ml aliquots at 0.3 mg/ml of lysate were incubated with an antibody-bead conjugate for ~2 hrs at 4°C. The unbound fractions were collected, and beads were subsequently washed three times with 1.0 ml of IP Buffer and processed for SDS-PAGE and immunoblot on 10-20% polyacrylamide gels. Prot-A: HRP was used as a secondary detection system (MilliporeSigma, cat. number P8651) at a dilution of 0.4 μg/ml for an experiment where a rabbit antibody was used for both immunoprecipitation and its corresponding Western Blot.

## Data Availability

Zenodo: Antibody Characterization Report for Midkine, https://doi.org/10.5281/zenodo.5644321. ^17^ Zenodo: Dataset for the Midkine antibody screening study, https://doi.org/10.5281/zenodo.7530472. ^18^ Data are available under the terms of the Creative Commons Attribution 4.0 International license (CC-BY 4.0).
