# Supplementary material for: A guide to selecting high-performing antibodies for human Midkine for use in Western blot and immunoprecipitation
Source: F1000Res. 2024 Aug 1;12:148. Originally published 2023 Feb 9. [Version 4] doi: 10.12688/f1000research.130587.4 (PMC11292187; doi:10.12688/f1000research.130587.4)
Supplement: Peer review history

# Reviewer report: Reviewer response for version 3

**DOI:** 10.5256/f1000research.169429.r306188

**Published:** 2024-07-31

**Authors:** Jens-Ulrich Rahfeld

**Affiliations:** 1 Fraunhofer Institute for Cell Therapy and Immunology, Department Molecular Drug Biochemistry and Therapy, Weinbergweg 22, 06120, Halle, Germany

Okay, no further comments! 

 Greetings

 Jens Rahfeld

Are sufficient details of methods and materials provided to allow replication by others?

Partly

Is the rationale for creating the dataset(s) clearly described?

Yes

Are the datasets clearly presented in a useable and accessible format?

Yes

Are the protocols appropriate and is the work technically sound?

Partly

Reviewer Expertise:

proteinchemisty

I confirm that I have read this submission and believe that I have an appropriate level of expertise to confirm that it is of an acceptable scientific standard.

# Reviewer report: Reviewer response for version 2

**DOI:** 10.5256/f1000research.160165.r269176

**Published:** 2024-05-13

**Authors:** Jens-Ulrich Rahfeld

**Affiliations:** 1 Fraunhofer Institute for Cell Therapy and Immunology, Department Molecular Drug Biochemistry and Therapy, Weinbergweg 22, 06120, Halle, Germany

The results of the publication are very helpful for the selection of suitable Midkine antibodies. 

 However, there are ambiguities in the experimental and methodological part: 

 The protocol mentions that the cell culture supernatants were obtained from starving cultures and then concentrated (also shown in scheme A in Figure 1.

 For the reproducibility of the preparation of these supernatants, there is no information on the cell density/ml of the cultivated HAP1 cells or the initial volume of the cell culture supernatant concentrated for analysis. 

 Furthermore, the description of Figure 1 B refers to cell lysates:

 " Lysates of HAP1 (WT and MDK KO) were prepared, and ~30 μg of protein was processed for Western blot."  

 This is not explained in the experimental section. This should definitely be explained or corrected.

Are sufficient details of methods and materials provided to allow replication by others?

Partly

Is the rationale for creating the dataset(s) clearly described?

Yes

Are the datasets clearly presented in a useable and accessible format?

Yes

Are the protocols appropriate and is the work technically sound?

Partly

Reviewer Expertise:

proteinchemisty

I confirm that I have read this submission and believe that I have an appropriate level of expertise to confirm that it is of an acceptable scientific standard, however I have significant reservations, as outlined above.

# Response

**Published:** 2024-07-19

**Authors:** Kathleen Southern

**Affiliations:** McGill University, Canada

Thank you to Dr. Jens-Ulrich Rahfeld for your comprehensive review of this article which presents as a guide to selecting high-performing antibodies for Midkine to progress research of this target associated to many pathological conditions. We hope our response to your comments (italicized) successfully answers any concerns you previously had.

***Reviewer Comment:***

*However, there are ambiguities in the experimental and methodological part:*

*The protocol mentions that the cell culture supernatants were obtained from starving cultures and then concentrated (also shown in scheme A in Figure 1.*

*For the reproducibility of the preparation of these supernatants, there is no information on the cell density/ml of the cultivated HAP1 cells or the initial volume of the cell culture supernatant concentrated for analysis.*

**Author Response:**

 To facilitate reproducibility of the antibody characterization platform utilized by the authors, we have included a reference to the full procedure, available on Protocol exchange ( https://doi.org/10.21203/rs.3.pex-2607/v1), to the new version (Version 3) of the article that will be submitted shortly. That being said, once the concentrated cell media is collected, the protein concentration is determined using Bradford reagent protein assay, and the concentrations are adjusted to ensure equal protein amounts are loaded into the gel. We understand the authors were not clear on this aspect of the sample preparation procedure for western blot and have included this to the Methods section in the new version of the article.

***Reviewer Comment:***

*Furthermore, the description of Figure 1 B refers to cell lysates:*

*" Lysates of HAP1 (WT and MDK KO) were prepared, and ~30 μg of protein was processed for Western blot."*

*This is not explained in the experimental section. This should definitely be explained or corrected.*

**Author Response:**

 To the “Antibody screening by Western blot on culture media” area of the methods section, we have clarified that 30 μg of protein was added to each lane of the polyacrylamide gel. We’ve also referenced our full detailed procedure, which includes troubleshooting when unprecedented results or issues arise.

# Response

**Published:** 2024-07-29

**Authors:** Kathleen Southern

**Affiliations:** McGill University, Canada

The authors would like to clarify our response to Jens-Ulrich Rahfeld in regards to  cell density and amount of protein used. 

 HAP1 cells (WT and MDK KO) were grown in 20 ml complete medium to 80% confluence in 150 mm dishes. At 80% confluency, HAP1 cells were at a density of ~1.0 x 10 ^6^/ml (~20.0 x 10 ^6^/ 20ml). To prepare for protein extraction, cells were washed 3x with PBS and starved for ~18 hrs. Culture media were collected and centrifuged for 10 min at 500 x g to eliminate cells and larger contaminants, then for 10 min at 4500 x g to eliminate smaller contaminants. Culture media were initially concentrated using Amicon Ultra-15 Centrifugal Filter Units (MilliporeSigma cat. number UFC9010) by centrifuging at 4000 x g for 15min. The resulting 500 μl of concentrated media from each filter unit were centrifuged again at 4000 x g for 15 min using Amicon Ultra- 0.5 Centrifugal Filter Units (MilliporeSigma cat. number UFC5010) to 200 μl. The final protein concentration was 0.3 mg/ml and 100 μl (30 μg) of media were used for each blot.

 An updated version of the manuscript has been submitted and we have included this protocol for cell preparation in the Methods section.

# Reviewer report: Reviewer response for version 2

**DOI:** 10.5256/f1000research.160165.r231070

**Published:** 2024-01-18

**Authors:** Jean-Pierre Bellier

**Affiliations:** 1 Department of Neurology, Brigham and Women's Hospital, Boston, MA, USA

I have no further comments.

Are sufficient details of methods and materials provided to allow replication by others?

Partly

Is the rationale for creating the dataset(s) clearly described?

Partly

Are the datasets clearly presented in a useable and accessible format?

Yes

Are the protocols appropriate and is the work technically sound?

Yes

Reviewer Expertise:

Neuroscience, antibody generation and use.

I confirm that I have read this submission and believe that I have an appropriate level of expertise to confirm that it is of an acceptable scientific standard.

# Reviewer report: Reviewer response for version 1

**DOI:** 10.5256/f1000research.143357.r208220

**Published:** 2023-11-29

**Authors:** Jean-Pierre Bellier

**Affiliations:** 1 Department of Neurology, Brigham and Women's Hospital, Boston, MA, USA

In this manuscript, Ayoubi et al. investigated the specificity of eight commercially available antibodies directed against the human Midkine protein. Midkine is a secreted pleiotropic protein involved in cell proliferation, survival, and the regulation of inflammation. It is often associated with tissue repair and cancer development and has also been shown to be present in amyloid-beta plaques in Alzheimer's disease.

 The manuscript is well-written, with experiments meticulously executed and technically sound, utilizing HAP1 cell WT and KO as controls. The availability of all datasets on Zenodo is highly commendable and professional.

 This work reveals that among the antibodies tested, three performed well for Western blotting (WB), and while all showed positive signals for immunoprecipitation (IP), six completely depleted the supernatant of the culture from Midkine protein. Two antibodies performed well in both experiments. Although the authors do not provide a detailed commentary and interpretation of their results, the data presentation is clear enough for readers to identify potentially suitable antibodies for WB and IP studies.

 Specific comments:

1. It would have been valuable if the authors had conducted a similar comparison for Immunofluorescence using WT and KO HAP1 cells.
2. For greater clarity, the authors should specify in the title that their study pertains to human Midkine and mention that HAP1 is a human cell line.
3. The term "high performing" used in the title may be misleading since the study primarily assesses antibody specificity rather than overall performance. If the authors want to use “High-performance” in the title, they should consider providing some interpretation of their data in the discussion.
4. In the introduction, the authors mentioned that Midkine (MK) is a potential biomarker and therapeutic target for Alzheimer's disease (AD). The original reference for the work demonstrating MK in senile plaques is "Yasuhara, O., Muramatsu, H., Kim, S. U., Muramatsu, T., Maruta, H., and McGeer, P. L. (1993) Midkine, a novel neurotrophic factor, is present in senile plaques of Alzheimer's disease. Biochem. Biophys. Res. Commun. 192, 246-251. [PMID: 8476427]"
5. Two of the antibodies featured in this study, NBP2-66948 and GTX116089, are discontinued. The authors should mention this.
6. Have the authors optimized the working concentration used? If so, they should explain the optimization process.
7. The authors should also provide a rationale for selecting the antibodies tested. Many of them are not commonly cited in the literature. An explanation for their choice of antibodies would enhance the manuscript's comprehensibility.
8. Finally, could the authors clarify why it is necessary to deplete the cells? Is it to promote Midkine secretion or to avoid interference from IgG in subsequent experiments? Further clarification on this point would be beneficial.
9. Some references (10-13) are not complete.

Are sufficient details of methods and materials provided to allow replication by others?

Partly

Is the rationale for creating the dataset(s) clearly described?

Partly

Are the datasets clearly presented in a useable and accessible format?

Yes

Are the protocols appropriate and is the work technically sound?

Yes

Reviewer Expertise:

Neuroscience, antibody generation and use.

I confirm that I have read this submission and believe that I have an appropriate level of expertise to confirm that it is of an acceptable scientific standard, however I have significant reservations, as outlined above.

# Response

**Published:** 2023-12-13

**Authors:** Kathleen Southern

**Affiliations:** McGill University, Canada

Dear Jean-Pierre Bellier,

 Thank you for your feedback. We have submitted a modified version of this manuscript, with your requested modifications included. We trust that the refinements made meet your expectations, enhance comprehensibility and address any concerns you might have had. Please see our responses to your specific comments below. 

**1. It would have been valuable if the authors had conducted a similar comparison for Immunofluorescence using WT and KO HAP1 cells.**

 We haven’t been able to detect intracellular Midkine by Western Blot. This is because once Midkine is synthesized into a protein, it is quickly secreted. Due to this factor, we expect that detecting intracellular Midkine via Immunofluorescence would be very difficult given the fixation and permeabilization methods used with our standard protocol.

**2. For greater clarity the authors should specify in the title that their study pertains to human midkine and mention HAP1 is a human cell line.**

 In the newly submitted version of this article,  the title has been updated to indicate that our study pertains to human Midkine. In the results and discussion section, we have clarified that the HAP1 cell line used is human. Thank you for this suggestion.

**3.The term "high performing" used in the title may be misleading since the study primarily assesses antibody specificity rather than overall performance. If the authors want to use “High-performance” in the title, they should consider providing some interpretation of their data in the discussion.**

 The new title of this article, “A guide to selecting high-performing antibodies for human Midkine for use in Western Blot and immunoprecipitation”. This modification aims to convey that the article’s objective is to guide researchers in selecting high-performing antibodies for their research’s needs. To the introduction, we have included a statement which indicates why the authors chose not to assess the results and draw conclusions on the antibodies performance. We trust these adjustments meet your expectations and prevent any further misinterpretations.

**4.In the introduction, the authors mentioned that Midkine (MK) is a potential biomarker and therapeutic target for Alzheimer's disease (AD). The original reference for the work demonstrating MK in senile plaques is "Yasuhara, O., Muramatsu, H., Kim, S. U., Muramatsu, T., Maruta, H., and McGeer, P. L. (1993) Midkine, a novel neurotrophic factor, is present in senile plaques of Alzheimer's disease. Biochem. Biophys. Res. Commun. 192, 246-251. [PMID: 8476427]"**

 Thank you for providing the original reference for the work done to isolate MDK in senile plaques. It has been included in the new version as reference number 7.

**5.Two of the antibodies featured in this study, NBP2-66948 and GTX116089, are discontinued. The authors should mention this.**

 This piece of information has been included in Table 2, where discontinued antibodies are now indicated with a superscript a.

**6.Have the authors optimized the working concentration used? If so, they should explain the optimization process.**

 This study for Midkine is part of a much larger collaborative initiative, seeking to characterize antibodies for all human proteins to address the antibody liability crisis. It would be very difficult to optimize every working parameter when in the process of trying to characterize antibodies for 20,000 proteins in the human proteome. That being said, we have developed universal protocols that has been successful for characterizing antibodies for 85 proteins thus far. In our experiments, a major parameter that we can control to optimize the process is the level of endogeneous protein in the selected cell type. We try to select cell lines, using DepMap portal, with adequate transcript levels of the protein target.

**7.The authors should also provide a rationale for selecting the antibodies tested. Many of them are not commonly cited in the literature. An explanation for their choice of antibodies would enhance the manuscript's comprehensibility.**

 The antibodies to be characterization under our standard procedure are donated by YCharOS partners ( https://ycharos.com/partners/). These industry partners cover nearly 28% of all antibodies listed on the Antibody registry ( antibodyregistry.org). When the industry partners are informed of upcoming targets, they donate the antibodies they have the capacity to supply at the time the study commences.

**8.Finally, could the authors clarify why it is necessary to deplete the cells? Is it to promote Midkine secretion or to avoid interference from IgG in subsequent experiments? Further clarification on this point would be beneficial.**

 To help us provide a complete response to this comment, could you specify what you mean by “deplete the cells”?

**9.Some references (10-13) are not complete.**

 The DOI for citations pertaining to our prior work, available on Zenodo, have been included in the new version submitted. While these references are not found on PubMed, and may seem “incomplete”, they are crucial in helping readers comprehend the protocols used and access the underlying data.
